# Genomic characterization of high-recurrence risk papillary thyroid carcinoma in a southern Chinese population

**DOI:** 10.1186/s13000-020-00962-8

**Published:** 2020-05-11

**Authors:** Min Li, Haitao Jia, Qiuqin Qian, Peng Wen, Chuan Chen, Yaqiong Hua, Kai Wang, Wenyong Zhang, Feng Shi

**Affiliations:** 1 Department of Nuclear Medicine, The Affiliated Cancer Hospital of Xiangya School of Medicine, Central South University, and Hunan Cancer Hospital, Changsha, 410013 China; 2 Shenzhen Cheerland Biotechnology Co., Ltd, Shenzhen, 518055 China; 3 School of Medicine, Southern University of Science and Technology, Shenzhen, 518055 China

**Keywords:** Papillary thyroid carcinoma, *BRAF*, *TERT*, *RET* fusion, *RAS*, TP53, Driver gene

## Abstract

**Background:**

The objective of this study was to investigate genetic variations and the relationships between these genetic variations and clinicopathological features of high-recurrence risk papillary thyroid carcinoma in a southern Chinese population.

**Methods:**

One hundred sixty-eight patients of high-recurrence risk papillary thyroid carcinoma were recruited for this study from 2017 to 2018. Formalin-fixed paraffin-embedded tissue and the data of clinicopathological characteristics were all collected and analyzed from these patients. We used next-generation sequencing technology to investigate the targeted gene mutations and gene fusions of the pathology specimens.

**Results:**

The frequency of candidate tumor driver gene mutation was 85.1% in 143 patients, including *BRAF* V600E mutation in 119 patients(70.8%), *RET* fusion in 13 patients(7.7%), *TERT* promoter mutations in 11 patients(6.5%), RAS (*HRAS*, *NRAS*, *KRAS*) gene mutations in 10 patients(6.0%), and other mutations involving *TP53*, *PIK3CA*, *AKT1*, *PTEN* and *NTRK1*. Concomitant presence of more than two genetic aberrations was seen in 27 patients (16.1%). Our study showed that *BRAF* V600E mutation is highly correlated with conventional PTC (*p* < 0.001), *BRAF* V600E and *TERT* promoter mutation duet was associated with older patient age (> 45, *p* = 0.003) and higher disease stage of III or IV (*p* = 0.002). *RAS* gene and *BRAF* V600E co-mutations were only seen in multifocal PTC (*p* = 0.015).

**Conclusion:**

In our high-recurrence risk PTC cohort, most patients had more than one driver gene aberration. Coexistence of *BRAF* V600E with *TERT* promoter mutations or with *RAS* mutations were significantly correlated with worse clinicopathological characteristics.

## Introduction

The incidence of thyroid cancer has increased throughout the world in the last few decades [1]. In the United States, its incidence has seen a 3.8-fold increase since 1973 [2], and in China, a study reported that more than a 3-fold increase in thyroid cancer incidence from 1983 to 2007 in Shanghai [3]. Papillary thyroid cancer (PTC) is the most common type of thyroid cancer and makes up about 85–90% of all thyroid cancer cases. Generally, PTC patients have a favorable prognosis with average 10-year survival of over 90%. However, recurrence remains relatively common, particularly for invasive PTC and cancer with *BRAF* V600E mutation [4].

A recent study of the molecular pathogenesis of PTC has revealed several genetic mutations that can be used as diagnostic markers as well as therapeutic targets [5]. The most common mutations in PTC, including *BRAF* point mutations, *RAS* point mutations, and *RET* gene rearrangements, perturb cell signaling in the mitogen-associated protein kinase (MAPK) pathway, leading to inappropriate cell growth and survival [6]. *BRAF* V600E mutation is the most common mutation seen in PTC, affecting approximately 50–60% of all PTC cases [7]. *BRAF* V600E mutation has been associated with more aggressive tumor characteristics, such as capsular invasion, lymph node metastasis, distal metastasis and recurrence [8]. *TERT* promoter mutations, most commonly C228T and C250T, have been associated with poor patient outcomes [9]. Although less frequent, mutations in PI3K/AKT pathway genes such as *PIK3CA,* and tumor suppressor genes such as *TP53* and *PTEN* have been identified in PTC*,* indicating complex genetic aberrations disturbing cellular growth and survival signals and contributing to the pathogenesis of PTC [10].

As thyroid cancer incidence has increased rapidly in China in recent years, and targeted therapies have become available in China to treat various types of cancer [11], we set out to characterize the genetic mutations of PTC in a high-recurrent risk cohort from Southern China to better understand the genetic-clinicopathologic correlation of this disease andprovide insight into the target therapy options.

## Material and Method

### Thyroid samples

One hundred sixty-eight patients of high-recurrence risk papillary thyroid carcinoma were recruited for this study from 2017 to 2018, They all had received radioiodine therapy. These patients were designated as the high-recurrence risk PTC group by clinical diagnosis of lymph node metastasis, capsular invasion or extrathyroidal invasion. Formalin-fixed paraffin-embedded (FFPE) tissue were collected from surgery at the Department of Pathology, Hunan Cancer Hospital. The ethics committee of Hunan Cancer Hospital passed ethical approval of this study, and the informed consents were confirmed by all participants before submitting this manuscript.

### DNA isolation

Genomic DNA were extracted from 15 × 5 μm thick tissue sections of FFPE tumor tissue using QIAamp DNA FFPE Tissue Kit according to the manufacturer’s instructions (Qiagen, Hilden, Germany). The percentage of tumor cells in the hematoxylin and eosin-stained slides were > 20% of the total tissue area, to ensure sufficient tumor DNA required for next generation sequencing. DNA concentrations were measured by a NanoDrop 2000 Spectrophotometer (Thermo Fisher Scientific, Waltham, MA, USA). All DNA concentrations were greater than 30 ng/L, and 100 ng DNA were used for NGS library construction.

### NGS library preparation

For NGS library preparation, DNA was fragmented using Covaris M220. Fragments of 200-400 bp in size were selected by beads (Agencourt AMPure XP kit; Beckman Coulter, Inc., Brea, CA, USA), then followed by end repair, phosphorylation and adaptor ligation. Then the library was pre-amplified with a high fidelity enzyme, followed by hybridization with a capture probe panel consisting of 14 PTC related genes (Supplementary 1), including 10 mutated genes (*BRAF*, *TERT*, *NRAS*, *HRAS*, *KRAS*, *PIK3CA*, *PTEN*, *AKT1*, *TP53*, *CTNNB1*) and 4 fusion genes (*RET*, *ALK*, *PAX8*, *NTRK1*), hybrid selection with magnetic beads and PCR amplification.

### Targeted DNA sequencing

After QC and quantification by Agilent 2100 Bioanalyzer (Agilent Technologies) and Qubit® 3.0 Fluorometer (Invitrogen), the capture-based targeted library were deep sequenced on NextSeq 500 (Illumina) with pair-end reads (2 × 150 cycles). The raw sequence data were mapped to the human genome (hg19) using BWA Aligner 0.7.10.

## Results

Tumor tissues from 168 cases of thyroid papillary carcinoma were analyzed by next-generation sequencing. The patients live in Southern China and of Han ethnicity. Thirty-seven were males and 131 were females. The average age of cancer onset was 38.8 years for males and 39.8 years for females. The general characteristics of the study population are summarized in Table 1. Most of the PTC patients were diagnosed with conventional PTC (92.9%, 156/168), the remaining patients were diagnosed with follicular variant PTC (7.1%, 12/168). The clinicopathological information of 168 patients were collected and shown in Supplementary Table 2.

**Table 1:** Clinical characteristics of 168 PTC patients in southern Chinese populations

Characteristics	*N* = 168
	No. (%)
Gender	
Female	131 (78.0)
Male	37 (22.0)
Age	
< 45	107 (63.7)
≥ 45	61 (36.3)
Subtypes	
conventional PTC	156 (92.9)
follicular variant PTC	12 (7.1)
Lymph node metastasis	
Yes	155 (92.3)
No	13 (7.7)
AJCC disease stage	
I + II	109 (64.9)
III + IV	59 (35.1)
Lesion number	
Single lesion	78 (46.4)
Multiple lesions	90 (53.6)

The frequency of candidate tumor driver gene mutation was 85.1% (143/168). The results showed that *BRAF* V600E was the most common mutation type in PTC with a mutation frequency of 70.8% (119/168). The next most frequent mutations in this patient population was *RET* fusion, which was seen in 7.7% (13/168) of patients. *TERT* promoter mutations C228T or C250T were found in 6.5% (11/168) of patients. RAS (*HRAS*, *NRAS*, *KRAS*) gene mutations had a frequency of 6.0% (10/168). Other mutations included *TP53*, *PIK3CA*, *AKT1*, *PTEN* and *NTRK1* fusion (Table 2).

**Table 2:** Genetic variants of 168 PTC patients in southern Chinese populations

Genetic Variants	This study (*N* = 168)	
	No.	%
*BRAF* V600E	119	70.8
Gene fusion status	15	8.9
*RET* fusion	13	7.7
*NTRK1* fusion	2	1.2
*TERT* status	11	6.5
C228T	10	6.0
C250T	1	0.5
RAS status	10	6.0
*NRAS*	4	2.4
*HRAS*	4	2.4
*KRAS*	2	1.2
*TP53* mutation	5	3.0
*PIK3CA* mutation	4	2.4
*AKT1* mutation	3	1.8
*PTEN* mutation	2	1.2

**Fig. 1:**
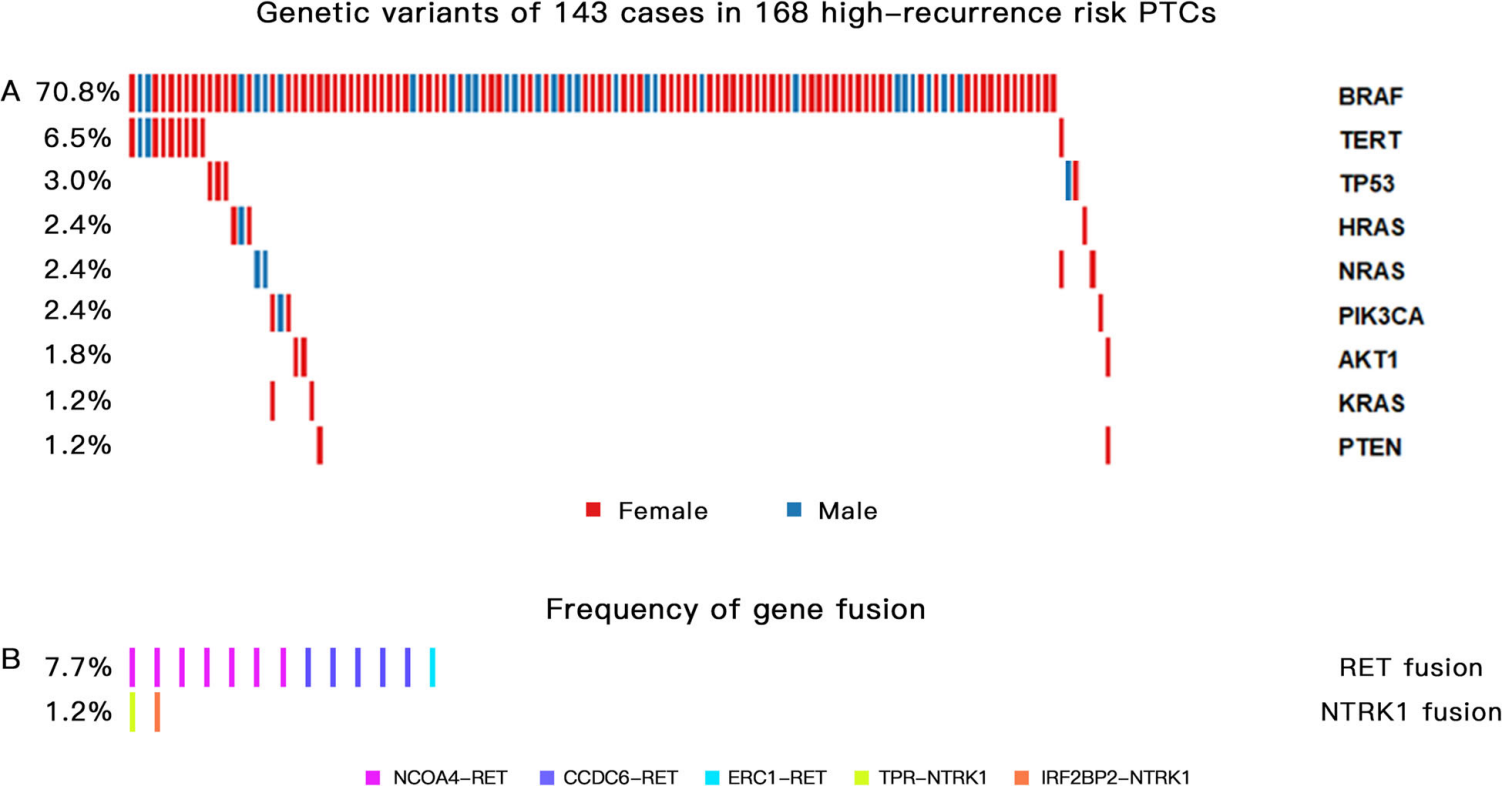
Genetic variants of 143 PTC patients in southern Chinese populations. One hundred forty-three PTC patients with driver gene mutation, including 128 cases with point mutation or promoter mutation, 15 cases with gene fusion. **a** Allelic frequency of *BRAF V600E* and other mutations in 128 patients with at least one mutation, gene fusion is excluded. **b** 3 types of *RET* fusion and 2 types of *NTRK1* fusion

In this study, fusion gene mutations were detected in 15 PTC cases (8.9%, 15/168), of which 13 involved *RET* fusions, and the remaining 2 cases involved *NTRK1* fusions. *NCOA4-RET* fusion was seen in 7 cases and *CCDC6-RET* was seen in 5 cases. *ERC1-RET* was seen in one case. *NTRK1* fusion mutations were seen in 2 cases. No *ALK* or *PAX8-PPARγ* fusions were detected. (Fig. 1).

Mutations in the *TERT* promoter region were the third most common mutation type in this study, primarily *TERT* C228T and C250T mutations. There were 10 cases with C228T mutation and 1 case with C250T mutation. The next most frequently mutated gene in our study was the RAS family genes, with 10 cases in total: *NRAS* (4 cases), *HRAS* (4 cases), *KRAS* (2 cases). Mutations in *TP53* (5 cases), *PIK3CA* (3 cases), *AKT1* (3 cases), *PTEN*(2 cases) were also detected in this study.

Twenty-seven cases (16.1%, 27/168) of co-mutation with *BRAF* V600E were identified in this study, including one patient with *BRAF* + *PIK3CA* + *KRAS* triple mutations. Types of mutation Included: *BRAF* V600E + *TERT* (10 cases), *BRAF* V600E + RAS (7 cases), *BRAF* V600E + *TP53* (3 cases), *BRAF* V600E + *PIK3CA* (3 cases), *BRAF* V600E + *AKT1* (2 cases), *BRAF* V600E + *PTEN* (1 case), respectively (Table 3).

**Table 3:** Coexistence gene mutation of PTC

Gene mutation combination	*N* = 168	
	No.	%
*BRAF* + *TERT*		
*BRAF* + *TER*T C228T	9	5.4
*BRAF* + *TERT* C250T	1	0.5
*BRAF* + *RAS*		
*BRAF* + *HRAS*	3	1.8
*BRAF* + *NRAS*	2	1.2
*BRAF* + *KRAS*	2	1.2
*BRAF* + *TP53*	3	1.8
*BRAF* + *PIK3CA*	3	1.2
*BRAF* + *AKT1*	2	0.5
*BRAF* + *PTEN*	1	0.5

## Discussion

Although PTC typically has a fairly good prognosis, approximately 30% of patients will experience disease progression or recurrence [12]. Studies have identified several genes, e.g. *BRAF*, *TERT*, RAS, *RET*, that play important roles in disease initiation or progression [10]. In our study, we chose to characterize the mutations in a cohort of high-recurrence risk PTC patients and examined the correlation between genetic mutations and clinicopathologic features. We found that *BRAF* V600E alone and co-mutations status of *BRAF + TERT*, *BRAF + RAS* showed correlation with age, disease stage and lesion number (Table 4).

**Table 4:** Relationships between *BRAF* V600E alone or *RET* fusion alone or *BRAF + TERT* or *BRAF* + RAS mutations and clinicopathological features in PTC patients

Characteristics	*BRAF V600E*			*RET* Fusion			*BRAF* + *TERT*			*BRAF* + *RAS*		
	Positive (*N* = 119)	Negative (*N* = 49)	*P*-value	Positive (*N* = 13)	Negative (*N* = 155)	*P*-value	Positive (*N* = 10)	Negative (*N* = 158)	*P*-value	Positive (*N* = 7)	Negative (*N* = 161)	*P*-value
Gender												
Female	92 (77.3)	39 (79.6)	0.746	9 (71.4)	122 (78.6)	0.428	7 (70.0)	124 (78.5)	0.530	6 (85.7)	125 (77.6)	0.614
Male	27 (22.7)	10 (20.4)		4 (28.6)	33 (21.4)		3 (30.0)	34 (21.5)		1 (14.3)	36 (22.4)	
Age												
< 45	73 (61.3)	34 (69.4)	0.324	11 (85.7)	96 (61.7)	0.102	2 (20.0)	105 (66.5)	0.003*	5 (71.4)	102 (63.4)	0.679
≥ 45	46 (38.7)	15 (30.6)		2 (14.3)	59 (38.3)		8 (80.0)	53 (33.5)		2 (28.6)	58 (36.6)	
Subtypes												
conventional PTC	116 (97.5)	40 (81.6)	< 0.001*	11 (85.7)	145 (93.5)	0.230	10 (100)	146 (92.4)	0.366	7 (100)	149 (92.5)	0.454
follicular variant PTC	3 (2.5)	9 (18.4)		2 (14.3)	10 (6.5)		0 (0)	12 (7.6)		0 (0)	12 (7.5)	
Lymph node metastasis												
Yes	107 (89.9)	42 (85.7)	0.434	12 (92.9)	137 (88.3)	0.668	9 (90.0)	140 (88.6)	0.893	6 (85.7)	143 (88.8)	0.800
No	12 (10.1)	7 (24.3)		1 (7.1)	18 (11.7)		1 (10.0)	18 (11.4)		1 (14.3)	18 (11.2)	
AJCC disease stage												
I + II	75 (63.0)	34 (69.4)	0.432	11 (85.7)	98 (63.0)	0.121	2 (20.0)	107 (67.7)	0.002*	5 (71.4)	104 (64.6)	0.711
III + IV	44 (37.0)	15 (30.6)		2 (14.3)	57 (37.0)		8 (80.0)	51 (32.3)		2 (28.6)	57 (35.4)	
Lesion number												
Single lesion	54 (55.5)	24 (75.5)	0.671	10 (76.9)	93 (60.0)	0.655	2 (20.0)	76 (63.9)	0.084	0 (28.6)	78 (62.7)	0.015*
Multiple lesions	65 (44.5)	25 (24.5)		3 (23.1)	62 (40.0)		8 (80.0)	82 (36.1)		7 (71.4)	83 (37.3)	

Values are presented as number (%). *p < 0.05. BRAF+RAS means BRAF+NRAS and BRAF+HRAS and BRAF+KRAS dual mutations together

*BRAF* V600E is a driver mutation that plays an important role in PTC diagnosis, prognosis and treatment method selection. Currently, many studies have shown that *BRAF* V600E mutation correlates with other factors of poor prognosis, including patient age, bigger tumor size, extracapsular invasion, multifocality, lymph node metastasis, distant metastasis and higher TNM stage [13–15]. Our study showed that *BRAF* V600E mutation is highly correlated with PTC tumor type (*p* < 0.001). However, it is not correlated with gender, age, lymph node involvement, AJCC disease stage (AJCC 7th Edition), or lesion numbers. Zhang et al. also reported in a study of Chinese PTC patients that 88.3% of conventional PTC patients had *BRAF* V600E mutation [16]. Liang Guo et al. reported the *BRAF* V600E mutation was not associated with cervical lymph node metastasis (LNM), but the *BRAF* V600E expression had shown significantly associated with cervical LNM [17]. Shu liu et al. reported correlation of *BRAF* V600E with extrathyroidal tumor invasion in a Chinese PTC population, however, the authors reported no correlation with other clinicopathological features [8]. These different findings might be due to variations in the study cohorts in terms of age distribution, histological variants of tumors, environmental factors and disease staging..

Mutations involving gene fusions in multiple cancers are considered driver events that lead to tumorigenesis, thus providing potential diagnostic markers or targets for precision treatment. We examined gene fusions with *RET* and *NTRK1* in our study. *RET/PTC* fusion is the most common type of gene fusions in PTC. *RET* fusion is considered an early event in PTC tumorigenesis. Radiation exposure has been shown to increase the risk of *RET/PTC* fusion [18]. Approximately 90% of reported *RET/PTC* fusions are *RET/PTC1* (*CCDC6-RET*) and *RET/PTC3* (*NCOA4-RET*) [19], consistent with our findings, which showed a RET fusion percentage of 92.3%(12/13).

*NTRK1* fusion with *TPM3, TPR or TFG* genes are oncogenic in PTC, patients with *NTRK1* gene fusion mutations often have a poor prognosis and tend to have younger age [20]. Under the control of the thyroid globulin promoter, *TPR*-*NTRK1* transgenic mice develop thyroid hyperplasia and papillary thyroid cancer [21]. We found two cases of *NTRK1* gene fusion mutations in our study, with *TPR* and *IRF2BP2* being the fusion partners. Liang et al. reported a case of *IRF2BP2*-*NTRK1* fusion in Chinese patients. It was shown that *IRF2BP2*-*NTRK1* fusion led to a higher expression of *NTRK1* tyrosine kinase structural domain [22]. In our current study, we did not see *NTRK1* fusion correlated with patient clinicopathologic features.

*TERT* promoter mutations are relatively common in PTC, affecting approximately 10% of all PTC, with C228T being the most dominant mutation and C250T mutations making up a smaller percentage [5]. *TERT* promoter mutations have been associated with aggressive tumor behaviors and worse prognosis in thyroid cancer [23]. In a large study of 1892 PTC patients, it was found that *BRAF* V600E and *TERT* promoter mutations coexist in 7.7% of all primary PTC [24]. While each type of mutation alone had a modest adverse effect, the double mutations were associated with much worse clinicopathologic outcomes, including extrathyroidal invasion, lymph node metastasis, distant metastasis, and disease recurrence [9]. In our study, we identified 11 cases with *TERT* promoter mutations, with 10 cases of C228T mutation, and 1 case of C250T mutation. Among 11 cases with *TERT* promoter mutations, 10 cases also had *BRAF* V600E mutation, and 1 case had *NRAS* mutation. In our study, *BRAF* V600E and *TERT* promoter mutations coexist in 6% of all PTC, in the same range as the previous report [24]. We found that *BRAF* V600E and *TERT* promoter mutation duet was associated with older patient age (> 45, *p* = 0.003) and higher disease stage of III or IV (*p* = 0.002).

In our cohort of high-recurrence risk PTC patients, we found multiple cases of dual mutations of *BRAF* V600E together with another mutation, including *TERT*, *RAS*, *TP53*, *PIK3CA*, *AKT1,* and *PTEN*. While mutation duet of *BRAF* V600E and *TERT* were most common, we unexpectedly identified 7 cases with *BRAF* V600E and *RAS* dual mutations. *RAS* mutations have been seen in several thyroid cancer types, including follicular thyroid cancer, poorly differentiated thyroid cancer, undifferentiated thyroid cancer and PTC [25]. Xing et al. reported that *RAS* mutation alone does not indicate malignancy in thyroid tumors [26]. However, thyroid cancer with dual mutations of *RAS* with *BRAF* V600E or *TERT* was associated with worse clinicopathologic outcomes [11, 27]. In our current study, dual mutations of *RAS* and *BRAF* V600E were only seen in multifocal PTC (*p* = 0.015).

In conclusion, in our study of high-recurrent risk PTC, we saw a high prevalence of *BRAF* V600E mutation (70.8%). *BRAF* V600E and *TERT* dual mutations were associated with older patient age (> 45) and higher disease stage. *RAS* and *BRAF* V600E dual mutations were also seen in this patient cohort and were associated with multifocal disease. In general, *RAS* and *BRAF* V600E mutations tend to be mutually exclusive, however, there have been reports of their coexistence in PTC [11, 28]. Whether their coexistence affects clinicopathologic outcomes of PTC remains to be studied further.

## Supplementary information

**Additional file 1.**

**Additional file 2.**

## Data Availability

The datasets used and/or analyzed during the current study are available from the corresponding author upon reasonable request.

## Abbreviations

PTC: Papillary Thyroid Carcinoma
FFPE: Formalin-fixed paraffin-embedded
MAPK: Mitogen-associated protein kinase
NGS: Next-generation sequencing
PCR: Polymerase Chain Reaction
QC: Quality Control
TNM: Tumor Node Metastasis
AJCC: American Joint Committee on Cancer
LNM: Lymph node metastasis
BRAF: B-type Raf kinase
TERT: Telomerase reverse transcriptase
TP53: Tumor protein p53
RET: Rearranged during transfection
PIK3CA: PI3K subunit p110alpha
PTEN: Phosphataseandtensinhomolog
NTRK1: Neurotrophic tyrosine kinase, receptor, type 1

## References

[1] Carling T, Udelsman R (2014). Thyroid Cancer. Annu Rev Med.

[2] Mao Y, Xing M (2016). Recent incidences and differential trends of thyroid cancer in the USA. Endocr Relat Cancer.

[3] Wang Y, Wang W (2015). Increasing incidence of thyroid cancer in Shanghai, China, 1983-2007. Asia Pac J Public Health.

[4] Huang Y, Qu S, Zhu G, Wang F, Liu R, Shen X (2018). BRAF V600E mutation-assisted risk stratification of solitary Intrathyroidal papillary thyroid Cancer for precision treatment. J Natl Cancer Inst.

[5] Cancer Genome Atlas Research N (2014). Integrated genomic characterization of papillary thyroid carcinoma. Cell..

[6] Chen H, Luthra R, Routbort MJ, Patel KP, Cabanillas ME, Broaddus RR (2018). Molecular profile of advanced thyroid carcinomas by next-generation sequencing: characterizing tumors beyond diagnosis for targeted therapy. Mol Cancer Ther.

[7] Xing M (2007). BRAF mutation in papillary thyroid cancer: pathogenic role, molecular bases, and clinical implications. Endocr Rev.

[8] Liu S, Zhang B, Zhao Y, Chen P, Ji M, Hou P (2014). Association of BRAF V600E mutation with clinicopathological features of papillary thyroid carcinoma a study on a Chinese population. Int J Clin Exp Pathol.

[9] Shi X, Liu R, Qu S, Zhu G, Bishop J, Liu X (2015). Association of TERT promoter mutation 1,295,228 C>T with BRAF V600E mutation, older patient age, and distant metastasis in anaplastic thyroid cancer. J Clin Endocrinol Metab.

[10] Xing M (2013). Molecular pathogenesis and mechanisms of thyroid cancer. Nat Rev Cancer.

[11] Huang M, Yan C, Xiao J, Wang T, Ling R (2019). Relevance and clinicopathologic relationship of BRAF V600E, TERT and NRAS mutations for papillary thyroid carcinoma patients in Northwest China. Diagn Pathol.

[12] Medas F, Canu GL, Boi F, Lai ML, Erdas E, Calo PG. Predictive Factors of Recurrence in Patients with Differentiated Thyroid Carcinoma: A Retrospective Analysis on 579 Patients. Cancers (Basel). 2019;11(9). 10.3390/cancers11091230. 10.3390/cancers11091230 PMC6770388 31443531

[13] Basolo F, Torregrossa L, Giannini R, Miccoli M, Lupi C, Sensi E (2010). Correlation between the BRAF V600E mutation and tumor invasiveness in papillary thyroid carcinomas smaller than 20 millimeters: analysis of 1060 cases. J Clin Endocrinol Metab.

[14] Li C, Lee KC, Schneider EB, Zeiger MA (2012). BRAF V600E mutation and its association with clinicopathological features of papillary thyroid cancer: a meta-analysis. J Clin Endocrinol Metab.

[15] Wada N, Masudo K, Nakayama H, Suganuma N, Matsuzu K, Hirakawa S (2008). Clinical outcomes in older or younger patients with papillary thyroid carcinoma: impact of lymphadenopathy and patient age. Eur J Surg Oncol.

[16] Zhang B, Xu CW, Wu YF, Man QH, Song YY, Wang JJ (2016). Diagnostic significance of the BRAF V600E mutation in conventional papillary thyroid carcinomas. Int J Clin Exp Med.

[17] Guo L, Ma YQ, Yao Y, Wu M, Deng ZH, Zhu FW (2019). Role of ultrasonographic features and quantified BRAFV600E mutation in lymph node metastasis in Chinese patients with papillary thyroid carcinoma. Sci Rep.

[18] Prescott JD, Zeiger MA (2015). The RET oncogene in papillary thyroid carcinoma. Cancer..

[19] Carlomagno F (2012). Thyroid Cancer: role of RET and beyond. Eur Thyroid J.

[20] Greco A, Miranda C, Pierotti MA (2010). Rearrangements of NTRK1 gene in papillary thyroid carcinoma. Mol Cell Endocrinol.

[21] Russell JP, Powel DJ, Cunnane M, Greco A, Portella G, Santoro M (2000). The TRK-T1 fusion protein induces neoplastic transformation of thyroid epithelium. Oncogene..

[22] Liang J, Cai W, Feng D, Teng H, Mao F, Jiang Y (2018). Genetic landscape of papillary thyroid carcinoma in the Chinese population. J Pathol.

[23] Jin L, Chen E, Dong S, Cai Y, Zhang X, Zhou Y (2016). BRAF and TERT promoter mutations in the aggressiveness of papillary thyroid carcinoma a study of 653 patients. Oncotarget.

[24] Liu R, Bishop J, Zhu G, Zhang T, Ladenson PW, Xing M (2017). Mortality risk stratification by combining BRAF V600E and TERT promoter mutations in papillary thyroid Cancer: genetic duet of BRAF and TERT promoter mutations in thyroid Cancer mortality. JAMA Oncol.

[25] Howell GM, Hodak SP, Yip L (2013). RAS mutations in thyroid cancer. Oncologist..

[26] Xing M (2016). Clinical utility of RAS mutations in thyroid cancer: a blurred picture now emerging clearer. BMC Med.

[27] Sohn SY, Park WY, Shin HT, Bae JS, Ki CS, Oh YL (2016). Highly concordant key genetic alterations in primary tumors and matched distant metastases in differentiated thyroid Cancer. Thyroid..

[28] Zou M, Baitei EY, Alzahrani AS, BinHumaid FS, Alkhafaji D, Al-Rijjal RA (2014). Concomitant RAS, RET/PTC, or BRAF mutations in advanced stage of papillary thyroid carcinoma. Thyroid..

