# Supplementary material for: Genomic characterization of high-recurrence risk papillary thyroid carcinoma in a southern Chinese population
Source: Diagn Pathol. 2020 May 11;15:49. doi: 10.1186/s13000-020-00962-8 (PMC7212670; doi:10.1186/s13000-020-00962-8)
Supplement: Supplementary file 1 — Additional file 1. [file 13000_2020_962_MOESM1_ESM.doc]

**Table S1.**

Target gene panel-Mutation hotspots

| Gene symbol | Exons |
| --- | --- |
| BRAF | 15 |
| NRAS | 2,3 |
| HRAS | 2,3 |
| KRAS | 3 |
| TERT | Promoter region |
| PIK3CA | All |
| PTEN | All |
| AKT1 | 3,4,10,11 |
| TP53 | All |
| CTNNB1 | 3 |

Target gene panel-Gene fusion

| Gene symbol |  |  |  |  |
| --- | --- | --- | --- | --- |
| RET | CCDC6 | NCOA4 | PCM1 | GOLGA5 |
| ERC1 | PPKAR1A | TRIM27 | TRIM24 | TRIM33 |
| KTN1 | HOOK3 | ALK | EML4 | STRN |
| PAX8 | PPARG | NTRK1 | TFG | TPM3 |
| TPR | IRF2BP2 |  |  |  |
